# Basic Regulatory Principles of *Escherichia coli'*s Electron Transport Chain for Varying Oxygen Conditions

**DOI:** 10.1371/journal.pone.0107640

**Published:** 2014-09-30

**Authors:** Sebastian G. Henkel, Alexander Ter Beek, Sonja Steinsiek, Stefan Stagge, Katja Bettenbrock, M. Joost Teixeira de Mattos, Thomas Sauter, Oliver Sawodny, Michael Ederer

**Affiliations:** 1 Institute for System Dynamics, University of Stuttgart, Stuttgart, Germany; 2 Molecular Microbial Physiology, Swammerdam Institute for Life Sciences, University of Amsterdam, Amsterdam, The Netherlands; 3 Experimental Systems Biology, Max-Planck-Institute for Dynamics of Complex Technical Systems, Magdeburg, Germany; 4 Life Science Research Unit, Université du Luxembourg, Luxembourg, Luxembourg; Universidad de La Laguna, Spain

## Abstract

For adaptation between anaerobic, micro-aerobic and aerobic conditions *Escherichia coli'*s metabolism and in particular its electron transport chain (ETC) is highly regulated. Although it is known that the global transcriptional regulators FNR and ArcA are involved in oxygen response it is unclear how they interplay in the regulation of ETC enzymes under micro-aerobic chemostat conditions. Also, there are diverse results which and how quinones (oxidised/reduced, ubiquinone/other quinones) are controlling the ArcBA two-component system. In the following a mathematical model of the *E. coli* ETC linked to basic modules for substrate uptake, fermentation product excretion and biomass formation is introduced. The kinetic modelling focusses on regulatory principles of the ETC for varying oxygen conditions in glucose-limited continuous cultures. The model is based on the balance of electron donation (glucose) and acceptance (oxygen or other acceptors). Also, it is able to account for different chemostat conditions due to changed substrate concentrations and dilution rates. The parameter identification process is divided into an estimation and a validation step based on previously published and new experimental data. The model shows that experimentally observed, qualitatively different behaviour of the ubiquinone redox state and the ArcA activity profile in the micro-aerobic range for different experimental conditions can emerge from a single network structure. The network structure features a strong feed-forward effect from the FNR regulatory system to the ArcBA regulatory system via a common control of the dehydrogenases of the ETC. The model supports the hypothesis that ubiquinone but not ubiquinol plays a key role in determining the activity of ArcBA in a glucose-limited chemostat at micro-aerobic conditions.

## Introduction

Microbial cells are able to adapt to different environmental conditions like temperature, pH, water activity, oxygen availability or substrate type. This requires reorganisation of the metabolism in order to reach short and long term adaptations. A quantitative and systems-level understanding of these processes will expand our insight of regulatory principles and can contribute to the elucidation of molecular mechanisms. Further, this knowledge can be employed in applied industrial settings, e.g. optimised production of organic compounds.

*Escherichia coli* is a facultative anaerobic microorganism, i.e. it can survive at various levels of oxygenation, [1], [2]. Those levels can be assigned to fully anaerobic, fully aerobic and intermediate micro-aerobic conditions. In the complete absence of oxygen (0% aerobiosis, anaerobiosis) or any other external electron acceptor the cell's fermentative pathways are active. Increased oxygen availability leads to the micro-aerobic (semi-aerobic) state which is an intermediate range where both fermentative and respiratory pathways are active. If the oxygen availability increases above a certain threshold no more fermentation products are excreted. Thus, full aerobiosis (100% aerobiosis) can be defined for the minimal oxygen inflow without any net production of fermentation products like acetate. As reported earlier, in glucose limited continuous cultures of *E. coli* the respective steady state acetate fluxes show a linear decrease to zero from 0% to 100% aerobiosis, [3]–[6]. This definition of the aerobiosis scale offers the possibility to get comparable measurement data across different experimental settings and laboratories. A limitation of this definition is that fully aerobic *E. coli* populations produce acetate at certain experimental conditions. This overflow metabolism occurs for high growth rates in the wild-type [7] and for some mutants (e.g. Δ*sdhC*) already at lower growth rates [8]. Therefore, the aerobiosis scale can be applied to micro-aerobic steady state experiments of glucose-limited continuous cultures of wild-type *E. coli* at low dilution rates.

Oxygen serves as a final electron acceptor of the electron transport or respiratory chain (ETC), [9, 10, and references therein]. The ETC's function is the successive transport of electrons from electron donors to electron acceptors while translocating protons from cytoplasm via the inner membrane into periplasmic space. The resulting proton gradient (proton motive force) may be used for ATP synthesis or for other energy consuming processes linked to the membrane, such as transport or flagellar motion. The central reactions of the aerobic *E. coli* ETC can be classified into categories: *Dehydrogenases* oxidise cytoplasmic electron donors, like NADH and FADH, by reducing membrane-associated quinones to quinoles. *Terminal oxidases* re-oxidise the quinoles using the external electron acceptor oxygen. The *E. coli* ETC uses the redox pairs ubiquinone/ubiquinol, menaquinone/menaquinol as well as demethylmenaquinone/demethylmenaquinol, [10]. For growth with oxygen as the sole electron acceptor, the most important enzymes are NADH-dehydrogenase I (Nuo) [11] and II (Ndh) [12], succinate-dehydrogenase (Sdh) and fumarate-reductase (Frd) [13], [14], as well as terminal oxidases cytochrome *bd*-I [15], cytochrome *bd*-II [16] and cytochrome *bo* [17]. Most of these enzymes differ in reactants, number of translocated protons, kinetic parameters and the range of activity within the aerobiosis scale. This allows for an adaptation to oxygen availability and due to a changed energy budget this leads to different substrate-biomass yields.

The oxygen response is mainly controlled by the global transcriptional regulators FNR and ArcA, [10]. The transcription factor (TF) FNR represses the gene expression of oxidases, [18], [19], and dehydrogenases, [20]–[23]. In case of ArcA, the situation is less distinct, because this TF represses *cyo*, *nuo* and *sdh*, but activates *cydAB*, *appBC* (*cyxAB*) and *ndh*, see [18]–[24]. While for FNR it is largely accepted that oxygen concentration is sensed directly [25]–[27], different mechanisms were suggested for the two-component system ArcBA. Amongst possible candidates for regulation of the sensor kinase ArcB, acetate, D-lactate, pyruvate and NADH have been proposed, [28]. In recent publications, the role of quinones was emphasised. There is substantial evidence that ArcB is repressed by oxidised quinones, [29]. However, the actual importance of the different quinone forms for the activity of ArcA is under debate. Bekker et al. [30] and Sharma et al. [31] showed that the *in vivo* ArcA activity is repressed by oxidised ubiquinones, menaquinones and demethylmenaquinones. Therefore, a mechanism was proposed by which oxidised quinones bind to ArcBA leading to a deactivation of the TF while reduced quinones reactivate ArcA. In contrast, Alvarez et al. [32] considered redox reactions and measured redox potentials between quinones and ArcBA. They propose a mechanism which describes the effect of deactivation by ubiquinone oxidising ArcB and reactivation by menaquinol reducing ArcB.

There are some dynamic modelling approaches describing electron transport chains. Jünemann et al. [33] investigated the catalytic cycle of cytochrome *bd* oxidase of *E. coli* and Beard [34] described a biophysical model of the respiratory system and oxidative phosphorylation of cardiac mitochondria. Recent publications presented a very detailed model of the *E. coli* electron transport chain focussing on the conformation of the different regulator species and genetic expression of single oxidases, [35]. This system's boundary was defined by the NADH/NAD couple and was intended to be integrated into a larger model also incorporating influences of other parts of metabolism. Ederer et al. [36] presented a model of the branched electron transport chain embedded into a model of the central metabolism. The model was used to describe the effect of the oxygen availability on the fluxes and concentrations in the central metabolism. For purple non-sulfur bacteria a model of the respiratory chain resembling that of *E. coli* was described taking into account also thermodynamic considerations, [37]. Further, a probabilistic model for *E. coli* ETC transcriptome dynamics was proposed, [38]. Considering the ETC in a broader context of balancing electron donors and acceptors, substrate uptake is another important subsystem of central metabolism. For growth on glucose Bettenbrock et al. [39] presented a phenomenological yield model of *E. coli* that was integrated into a larger model for investigation of catabolite repression.

Here, we present and investigate a mathematical modelling approach that provides an integrated description of the reactions of the ETC, the activity of the transcription factors ArcA and FNR and of substrate uptake, growth and fermentative pathways. The model is able to describe and explain the behaviour of ubiquinone/ubiquinol and ArcA activity for steady state continuous cultures in dependence on the aerobiosis value. In particular, it explains how qualitatively different behaviour emerges from the same network structure under different experimental conditions. The parameter identification contains a validation step and accounts for possible differences in gene expression of two different wild-type strains. The model structure of the ETC features a feed-forward path from the FNR feedback regulatory system to the ArcA feedback regulatory system. The model strongly suggests that the inactivation of ArcA by ubiquinone determines the activity of ArcA for the studied conditions and thus contributes to the above mentioned debate.

## Results

In the results section the mathematical model is derived, parameters determined and model simulations compared with measurement data. Some aspects of the model, several parameter values and the comparison of data are based on two experimental setups “ExpA” and “ExpB”. They both constitute micro-aerobic steady state experiments of glucose-limited continuous cultures of wild-type *E. coli* at low dilution rates. However, the experimental setups differ in some experimental conditions, see Table 1. While the model addresses differences in dilution rate, glucose concentration and to some extent the strain, differences in temperature and pH are assumed to have a minor effect. Eventually, Table 2 gives an overview of all employed data.

**Table 1:** Comparison of the experimental conditions “ExpA” and “ExpB”.

Variable	“ExpA”	“ExpB”
Strain	MC4100	MG1655
*D*	0.15 h^−1^	0.2 h^−1^
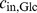	45 mM	20 mM
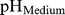	7	6.9
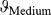	35°C	37°C

Table contains information about differences in strain, dilution rate *D*, glucose concentration in the feed 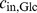 as well as pH and temperature 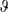 in the medium.

**Table 2:** Overview of the displayed measurement data and their origin.

Symbol	Variable	Figure(s)	Origin
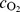	Oxygen concentration	4 A	[3, p.68], [5], [6]
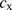	Biomass	4 B	[3, p.156], [6], [8], this pub.
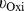	Oxygen uptake rate/Oxidase flux	4 C	[3, p.158], [6], [8], this pub.
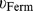	Fermentation products flux	4 D	[4], [8], this pub.
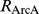	ArcA activity	5 A	[3, p.141], [30], [60], [53]
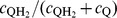	Quinone redox state	5 B	[30], [60], this pub.
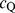	Quinone concentration	5 C	[60], this pub.
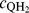	Quinol concentration	5 D	this pub.

### Modelling

**Figure 1:**
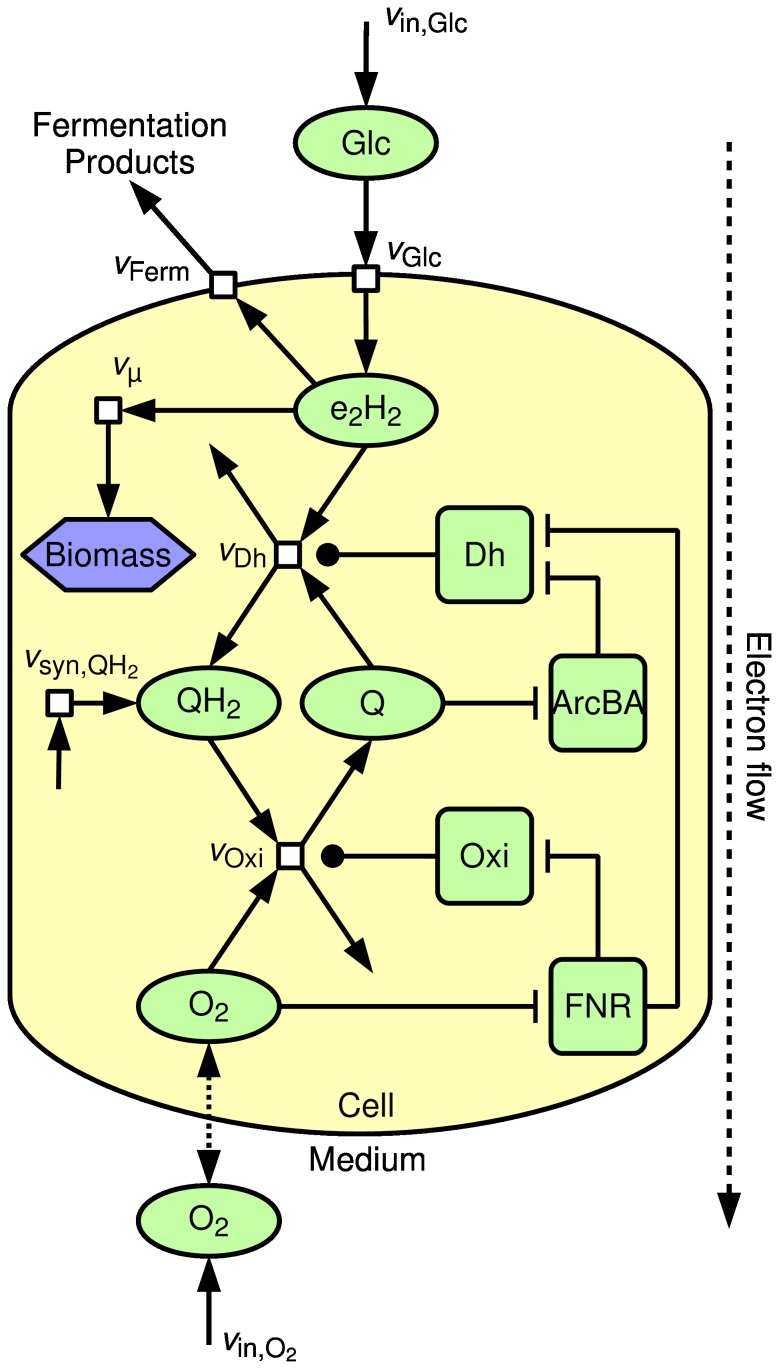
Model structure of the electron transport chain in *E. coli*. Glucose Glc is taken up (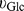) and creates a reducing potential e_2_H_2_ (pool of available electron pairs). This pool is connected to a dehydrogenase-catalysed reaction 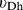 reducing quinone Q to quinol QH_2_. Quinol is oxidised in an oxidase-catalysed reaction 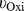 reducing oxygen O_2_ and can also be synthesised *de novo* (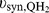). The ETC reaction enzymes (Oxi, Dh) are regulated by FNR and ArcBA. The reducing potential also supply reactions for forming biomass (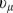) and fermentation products (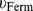).

Fig. 1 shows the structure of the presented model, containing the electron transport chain (ETC) as well as basic modules for glucose uptake, product excretion and biomass formation. Both glucose (electron donor) and oxygen (electron acceptor) are taken up by the organism. The terminal oxidase transfers electrons from quinol to oxygen, thereby oxidizing quinol to quinone. The dehydrogenase reduces quinone to quinol by transferring cytoplasmic electron pairs “e_2_H_2_”. The pool variable “e_2_H_2_” comprises all electron pairs that directly or indirectly can be transferred to electron acceptors. Most metabolites contribute to this pool. For example, glucose carries 12 electron pairs (C_6_H_12_O_6_+6O_2_→6CO_2_+6H_2_O) and pyruvate carries 5 electron pairs (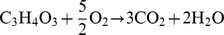), see also Text S1. Metabolites that carry electron pairs can be either used to reduce quinones, excreted (for example during mixed acid fermentation) or incorporated into cell constituents contributing to dry cell weight (biomass). ETC enzymes are regulated by the global transcriptional regulators FNR and ArcBA. Further, there is a reaction for *de novo* synthesis of quinol.

Since we were interested primarily in how ArcA and FNR react on the balance between electron acceptors and donors, we made the following simplifications: 1. The complex metabolic network of central metabolism was reduced to a single pool variable e_2_H_2_ that describes available electron pairs, 2. Different terminal oxidases and dehydrogenases of the ETC were combined into one flux, respectively, 3. Gene regulation and expression was described in a simplified manner, and 4. Different quinone species (ubiquinone, menaquinone, demethylmenaquinone) of each of the two forms (oxidised, reduced) were pooled together in one quinone and one quinol variable, respectively.

#### State variables

The model consists of ordinary differential equations for eight state variables, Eqs. (1a)–(1h): 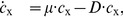
(1a)

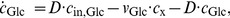
(1b)

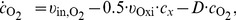
(1c)

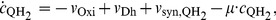
(1d)

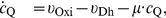
(1e)

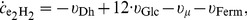
(1f)

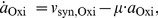
(1g)

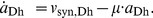
(1h)


The biomass concentration *c*_x_ in g_DCW_⋅L^−1^ (1a) is dynamically determined by growth *μ* and dilution *D*. The microorganisms have access to substrate, the extracellular glucose concentration *c*_Glc_ in mM (1b) being the electron donor, and to the extracellular (di)oxygen concentration 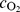 in mM (1c) being the electron acceptor provided by the aerated medium. The reduced form quinol 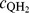 in 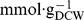 (1d) and the oxidised form quinone 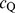 in 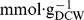 (1e) couple oxidase and dehydrogenase reactions. Throughout this document, the quinone redox state is defined by 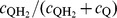. The reducing or electron potential 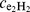 in 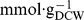 (1f) represents available electrons pairs. As part of the respiratory chain's regulatory loops, maximal enzyme activities of the oxidases 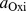 in 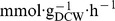 (1g) and the dehydrogenases 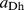 in 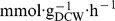 (1h) are influencing the respective reaction rates.

#### Biomass

In equation (1a) the terms 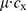 and 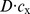 determine the change of biomass (dry cell weight) in 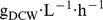. While the (specific) growth rate 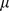 in 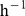 is dynamically changing depending on the glucose-biomass yield, the dilution 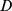 in h^−1^ ( = feed of the chemostat) was fixed by experimental conditions. Its value was 0.15 h^−1^ for “ExpA” and 0.2 h^−1^ for “ExpB”. In steady state 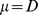 and 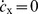.

#### Glucose uptake

The glucose change (1b) is expressed in 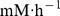. Glucose is fed into the chemostat by 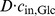 with the preset glucose concentration 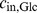 in the influent. For “ExpA” the value for this parameter was 45 mM and for “ExpB” 20 mM. The glucose uptake 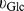 in 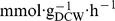 was defined by 
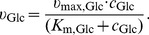
(2)applying irreversible Michaelis-Menten kinetics. Consistent with measurement data the maximal velocity was set to a value of 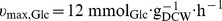. For the Michaelis constant we chose 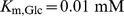 which was reported to be the affinity's magnitude of the relevant glucose transporters under chemostat conditions, PtsG and MglBAC, see [40]. The last term of equation (1b) denotes glucose dilution 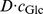.

#### Oxygen input and ETC reactions

The state equation of oxygen (1c) results from three fluxes expressed in mM⋅h^−1^: (i) oxygen inflow 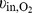 into the chemostat, (ii) oxygen uptake, i.e. consumption at the oxidase 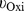, and (iii) dilution of oxygen 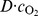. The inflow 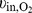 describes *net* inflow of oxygen that is the difference of oxygen in the input and the output gas. This variable was considered to be the input variable for investigation of the system behaviour. As proposed earlier, [3], [5], a linear relationship between oxygen input and aerobiosis *a* can be applied: 
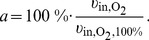
(3)


Oxygen uptake into the system 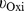 is directly connected with the oxidase-catalysed ETC reaction 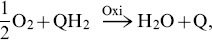
thereby changing the concentrations of quinol and quinone, (1d) and (1e), respectively. The rate equation was modelled by 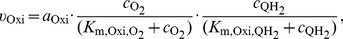
(4)i.e. for the oxidase an irreversible Michaelis-Menten kinetics with respect to oxygen and quinol as well as a variable maximal enzyme activity 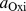 was applied. The term 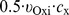 as part of oxygen's state equation (1c) refers to dioxygen O_2_ while the internal oxidase (4) is expressed in 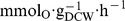. The Michaelis constants of different oxidases, i.e. the affinities to oxygen, have been reported to differ by two orders of magnitude. Values known so far include 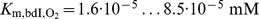, 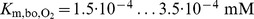, [41]–[43], and 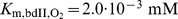, [44]. In the model there is only one unifying parameter 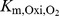 which was determined within the magnitude of literature values. Also, the parameter 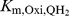 was determined by parameter identification.

Furthermore, the quinone pool is reduced by a dehydrogenase: 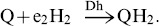



Similar to the oxidase reaction (4) the dehydrogenase flux was modelled by Michaelis-Menten kinetics 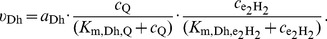
(5)


The two kinetic parameters were determined by parameter identification.

#### Growth

**Figure 2:**
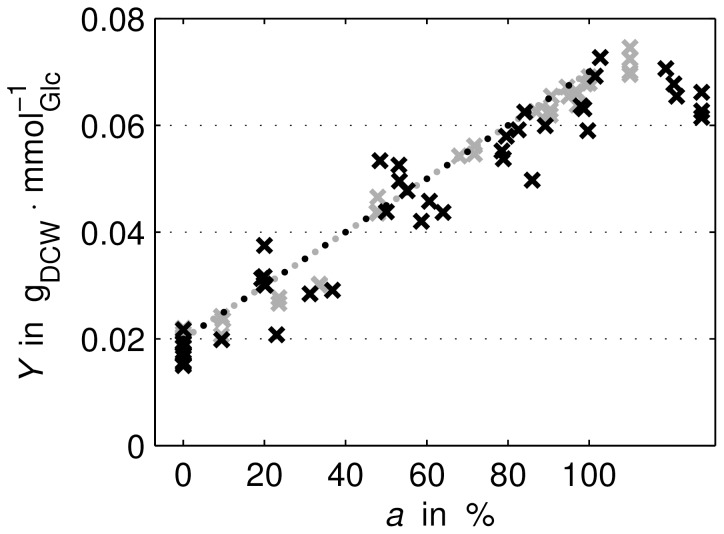
Comparison of calculated and measured glucose-biomass yield *Y* over aerobiosis *a*. Measurements: “ExpA” (grey crosses) and “ExpB” (black crosses). Calculation (dotted line): course can be calculated from *Y*_0%_ and *Y*_100%_ assuming a linear relationship with respect to aerobiosis, see (6) and (7).

The glucose-biomass yield depends strongly on oxygen uptake, see Fig. 2. To account for the varying yield coefficient, a simplified growth model including a phenomenological yield description was applied. First, we defined a variable 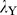 as follows: 
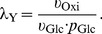
(6)


This variable is based on the ratio between oxygen flux (4) and glucose flux (2), i.e. electron acceptor and electron donor, scaled by the parameter 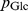 for the glucose proportion contributing to the yield. The parameter 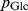 could be calculated from measurement data, see Text S2. Indeed, 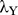 is proportional to aerobiosis and model parameters were chosen in a way that it is 1 for the fully aerobic state. Subsequently, this variable (6) was used in the phenomenological formula for calculating the yield 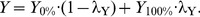
(7)


This linear relationship was motivated by measured glucose-biomass yield over aerobiosis, see Fig. 2. Experimental data were used to determine the parameters of the yield's bounds *Y*_0%_ and *Y*_100%_ for the anaerobic and aerobic state, respectively, see Text S2. Finally, the specific growth rate, 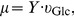
(8)can be calculated by using yield (7) and glucose flux (2).

#### Reducing potential e_2_H_2_ (electron pairs)

Glucose is the sole external electron donor and oxygen the sole electron acceptor under the studied experimental conditions. Depending on the oxygen availability, electrons originating from glucose are used for growth as well as converted into fermentation products or fed into the electron transport chain.

In the state equation for the pool of electron pairs 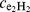 (1f) the second term 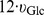, see also (2), describes that one molecule of glucose that is taken up carries 12 electron pairs. The third flux 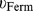 denotes the change of e_2_H_2_ due to excretion of metabolites carrying electron pairs, for example during mixed acid fermentation. For this flux, we assumed a simplified description depending on the amount of electron pairs: 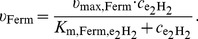
(9)


The simulated rate should reproduce the total flux of measured fermentation products 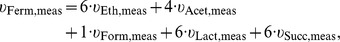
which could be calculated from individual components, see Text S1. While for (9) the Michaelis constant 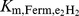 was determined by parameter identification, the maximal velocity of fermentation product excretion was set to the mean rate of “ExpB” at 0% aerobiosis, 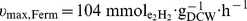, being the higher value of both experimental conditions.

The term 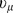 of (1f) represents the flux of electron pairs incorporated into biomass by formation of cell constituents. We assumed that 1 g dry biomass carries *X_μ_* electron pairs (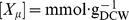) and write 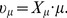
(10)


For parameter identification the lower and upper bound of parameter *X_μ_* could be estimated from the e_2_H_2_ state equation (1f) in steady state applying measurement data, see Text S2.

#### ETC regulatory loops

The ETC model is completed by equations describing the enzyme synthesis controlled by the global transcriptional regulators FNR and ArcBA. Dimerized FNR is the active form of the TF and its activity is regulated directly by oxygen, [25]–[27]. ArcBA is a two-component regulatory system consisting of the ArcB sensor-kinase and the TF ArcA. The active form ArcA-P is a phosphorylated octamer. Here, we investigated quinones as one of the proposed regulators mentioned in the introduction. For describing the activities of FNR and ArcAB, we used a simplified approach and introduced activity variables *R*_FNR_ and *R*_ArcA_ which can be regulated between 0 and 1 according to the following phenomenological Hill-equations: 
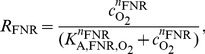
(11a)

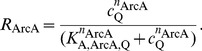
(11b)


**Figure 3:**
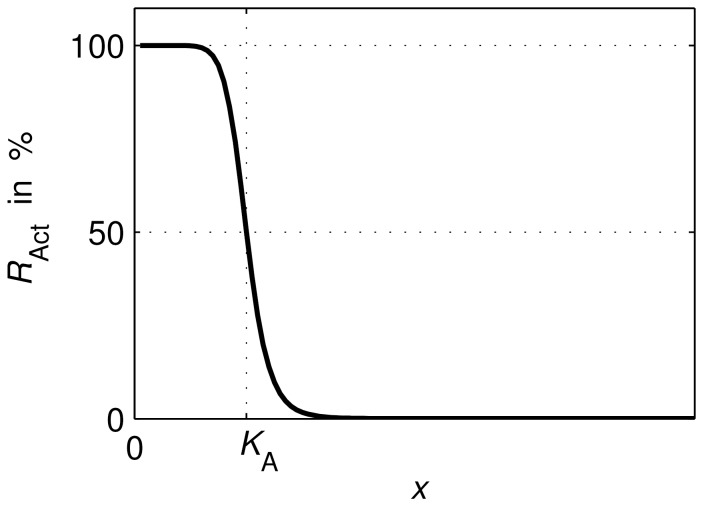
Transcription factor activity *R*_Act_ (e.g. FNR) in dependence on the respective metabolic signal *x* (e.g. O_2_). As modelled in equations (11a) and (11b), small changes of the metabolite in the range around *K*_A_ lead to a hypersensitive large change in the regulator activity.

Depending on the absolute value of the negative Hill coefficients *n*_FNR_ and *n*_ArcA_ a more or less switch-like behaviour of TF activities with respect to their repressing signals can be modelled, Fig. 3. Eventually, in a certain range small changes in a regulating metabolite lead to a hypersensitive large change in TF activity. Identified values of the Hill coefficients indicate whether the behaviour of the system depends on such a sensitive regulation. The repressing signals in (11a) and (11b) for FNR and ArcA are oxygen and quinone, respectively. It will be discussed later, why the latter formula depends on quinone and not also on quinol. The parameters of (11a) and (11b) were determined by parameter identification.

In *E. coli*, expression of the ETC oxidases and dehydrogenases depend on ArcA and FNR. The synthesis rates were modelled by 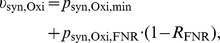


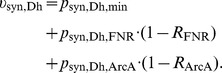



On the right hand side the 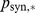 are free parameters for a constitutive (minimal) and regulator activity dependent synthesis. Inhibition of the synthesis rates by the regulators, (11a) and (11b), was modelled by 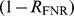 and 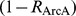, respectively. In equations (1g) and (1h) the second terms 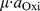 and 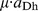 describe dilution of the enzymes due to cellular growth with the specific growth rate 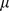.

#### Quinone synthesis

In measurement data, it was observed that also the overall content of ubiquinones 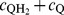 increases with aerobiosis, [30], which means that *de novo* synthesis depends on the aerobiosis value. Therefore, a quinone synthesis 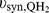 was introduced in (1d). The synthesis directly influences quinol as the end product, [45], but was not modelled in a detailed way here. Instead, in agreement to the measurements, we applied a simple synthesis equation depending linearly on 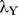 which itself increases linearly from 0 to 1 over the aerobiosis scale 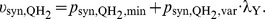



### Parameters and Simulation

#### Parameter types

The model contains different parameter types. A first type of parameters describes the different environmental or experimental conditions. Further, values or at least bounds for some other parameters could be taken from literature. The values of the next group of parameters could be calculated directly from measurement data, see Text S2. Finally, there are parameters, especially the ones for the synthesis equations that were determined applying an automatic parameter identification based on measurement data. An overview about the parameters, their final values and their origin can be found in Tables 3 and 4.

**Table 3:** Parameter overview I: values, units and references (origin).

Parameter	Value	Unit	Origin
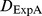	0.15	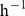	Experimental condition
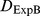	0.2	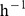	Experimental condition
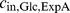	45	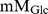	Experimental condition
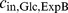	20	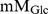	Experimental condition
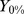	0.02	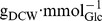	Measurement
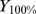	0.07	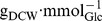	Measurement
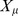	90.72	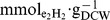	Measurement/Identification
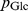	5.42	-	Measurement
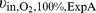	18.28	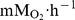	Measurement
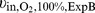	10.84	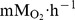	Measurement
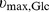	12	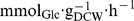	Measurement
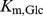	0.01	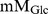	Literature
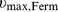	104	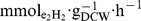	Measurement
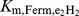	5.98	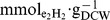	Identification
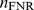	−10	-	Identification
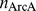	−10	-	Identification
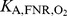	0.36⋅10^−3^	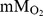	Identification
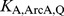	0.47⋅10^−3^	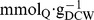	Identification
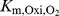	0.13⋅10^−3^	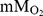	Literature/Identification
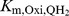	0.01⋅10^−3^	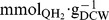	Identification
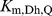	0.34⋅10^−3^	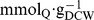	Identification
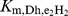	0.02	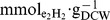	Identification

**Table 4:** Parameter overview II: Comparison of parameters for genetic regulation between experimental conditions “ExpA” and “ExpB”.

Parameter	Value “ExpA”	Value “ExpB”	Origin
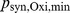	1.1933	Value “ExpA”	Identification
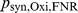	1.0337	1.9334	Identification
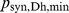	1.9500⋅10^−5^	Value “ExpA”	Identification
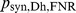	2.7747	3.5747	Identification
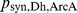	1.7467	3.4760	Identification
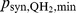	1.1473⋅10^−4^	Value “ExpA”	Identification
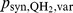	3.0780⋅10^−6^	4.2725⋅10^−4^	Identification

Parameter identification and model validation was based on the data of two experimental set-ups, “ExpA” and “ExpB”, see Table 1. Both data sets include measurements from micro-aerobic steady states of glucose-limited continuous cultures of wild-type *E. coli* at low dilution rates, see Table 2.

#### Parameter identification

**Figure 4:**
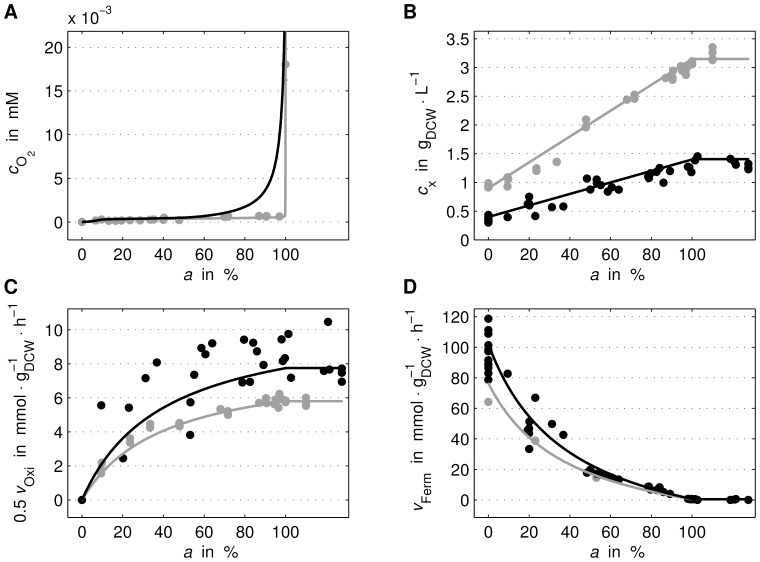
Comparison I of measured and simulated (lines) data. For the two experimental conditions “ExpA” (grey) and “ExpB” (black), the courses of oxygen concentration (A), biomass (B), oxygen uptake rate (C) and total fermentation flux (D) over aerobiosis *a* are shown.

**Figure 5:**
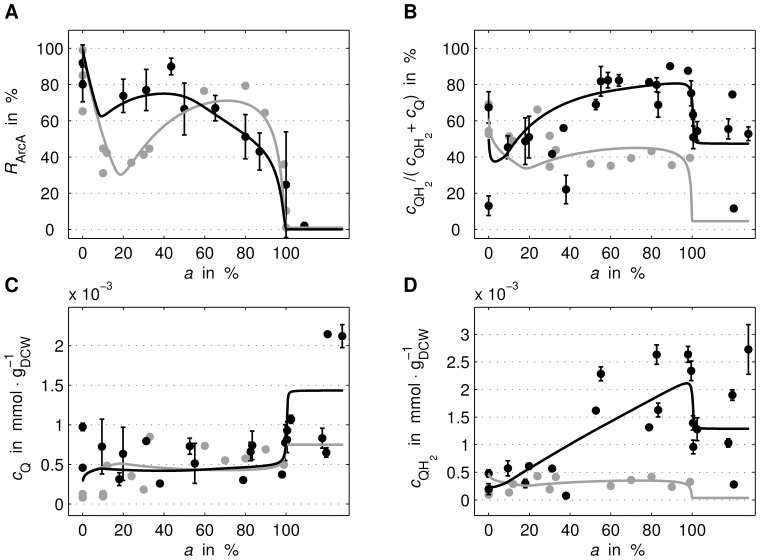
Comparison II of measured and simulated (lines) data. For the two experimental conditions “ExpA” (grey) and “ExpB” (black), the courses of ArcA activity (A), quinone redox state (B), (oxidised) quinone concentration (C) and quinol concentration (D) over aerobiosis *a* are shown. The experimental data *c*_Q_ and 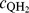 refer to ubiquinone and ubiquinol, respectively.

We used the experimental data set “ExpA” for identification of unknown parameter values. The fitted model describes the available measurement data well (see Figs. 4 and 5). The most noticeable identified parameter values are: (i) large negative values of the Hill exponents 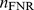 and 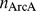 which hint at a sensitive regulation of the TFs by their metabolic signals and (ii) the Michaelis constant of the oxidase 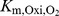 stays within its literature derived bounds.

#### Model validation

In order to validate the model, we applied the model fitted by data from “ExpA” to the experimental conditions “ExpB”. Since the experimental conditions feature different wild-type strains we assumed that most parameter values persist but that parameters related to gene expression can be different. Therefore, for “ExpB” we only re-identified parameters of gene expression and *de novo* synthesis of quinone and kept the previously identified values for the remaining majority of parameters. Noticeably, in both “ExpA” and “ExpB” the control of dehydrogenase synthesis by FNR (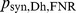) is stronger than the control of oxidase synthesis by FNR (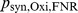). Further, in “ExpA” the regulation of dehydrogenase by FNR (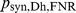) is stronger than by ArcA (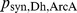). Finally, the variable quinone synthesis parameter 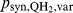 of “ExpB” is two orders of magnitude higher than of “ExpA”.

#### Comparison of measurements and simulation

For both experiments, simulation and experimental data were compared at different aerobiosis levels, see Figs. 4 and 5. Although for some variables of “ExpB” measurement data originated from different laboratories, due to comparability by aerobiosis calibration, those data are not discriminated in the figures. The additional Figure 6 shows predicted courses of the oxidase and dehydrogenase activity as well as the FNR activity for both experiments. In the following, the behaviour of the different variables, in particular differences between the two experimental set-ups or special features, is described based on the simulated courses.

**Figure 6:**
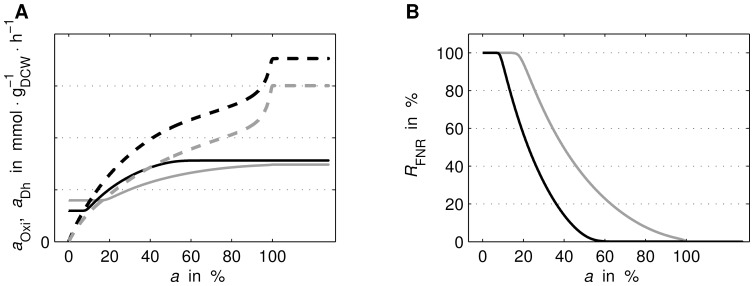
Predicted courses of enzyme activities and FNR. Simulated (predicted) maximal oxidase activity (continuous line) as well as maximal dehydrogenase activity (dashed line) (A) and FNR activity (B). For the two experimental conditions “ExpA” (grey) and “ExpB” (black) the courses over aerobiosis *a* are shown.

By comparison, the absolute oxygen level (dissolved oxygen), Fig. 4A, has a similar magnitude for both experimental conditions. A difference can be observed close to 100% aerobiosis. While for simulated “ExpA” there exists a very steep increase, “ExpB” shows a more continuous increase. Biomass concentration in “ExpA” is higher and increases stronger with oxygen availability than in “ExpB”, see Fig. 4B. In contrast, the biomass-specific oxygen uptake flux, Fig. 4C, is higher in “ExpB”. The total biomass-specific fermentation flux 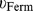 composed of individual fermentation fluxes (ethanol, acetate, formate and lactate as well as succinate) is lower in “ExpA”, see Fig. 4D.

Large differences between the two experimental conditions can be seen in ArcA regulator activity and quinones (ubiquinone and ubiquinol) including their redox state, see Fig. 5. Even though the expected behaviour of ArcA – maximal activity anaerobically, minimal activity aerobically – occurs for both experimental conditions, courses differ in the micro-aerobic range. While for “ExpA” the course exhibits a local minimum around 20% aerobiosis and another local maximum around 80% aerobiosis, the respective course of “ExpB” is not showing this pronounced behaviour, see Fig. 5A. For the latter data, the measurement exhibits an almost monotonous decrease and the simulation predicts two weak local extrema in the lower micro-aerobic range. Also, the behaviour of the quinones differ between the two experiments. The redox state level and the quinol concentration is much higher for “ExpB”, especially in the upper micro-aerobic range, see Figs. 5B and 5D. Further, the “ExpB” quinol data show a high variation around 100% aerobiosis. Looking more closely at the *simulated* quinone concentrations it can be observed that the courses are not monotonously increasing over the whole range but exhibiting very weak local extrema, see Fig. 5C.

Fig. 6 displays predicted courses for the oxidase and dehydrogenase enzyme activities as well as the FNR activity. The most prominent difference can be observed in the proposed oxidase enzyme activity. The simulated “ExpB” course changes between 10% and 50% aerobiosis only, see Fig. 6A. In the same range a larger decrease in simulated “ExpB” FNR activity can be observed, see Fig. 6B.

## Discussion

The mathematical modelling was motivated by three aspects: 1. The model should reproduce steady state data of a continuous culture with different experimental conditions (oxygen, glucose concentration, dilution rate). 2. The model should allow investigating oxygen-dependent regulation of the ETC, especially focussing on the control of ArcA by quinone(s). 3. The model should allow to explain the differences between two seemingly contradictory experimental data sets (“ExpA” and “ExpB”). Our goal was to develop a model that is as simple as possible but can contribute to the above aspects.

### Composition of the model

Therefore, a model of the regulated ETC was coupled with an electron balance and a phenomenological growth model. The applied growth model, equations (6)–(8), is able to account for changed experimental parameters of dilution rate and substrate concentration. The proposed linear description of the glucose-biomass yield is justified by measurement data for this variable, see Fig. 2.

A key aspect of the model is the introduction of the pool variable e_2_H_2_ that subsumes all electron pairs directly or indirectly available for respiration. This variable couples pathways that provide electrons (glucose uptake) and pathways that consume electrons (oxygen uptake, fermentation product excretion and growth). The good fit of simulation and measurements of the respective variables (Figs. 4B–D) for “ExpA” and “ExpB” demonstrates the validity of introducing the pool variable e_2_H_2_.

The model does not distinguish between the different oxidases (Cyo, Cyd, AppBC), dehydrogenases (Nuo, Ndh,…) and quinone species (ubiquinone, menaquinone, demethylmenaquinone) but considers only one oxidase activity, dehydrogenase activity and one type of quinones, respectively. This is a strong simplification because it neglects the different kinetics and energetics of the different enzymes and quinones. However, the simplification preserves basic constraints on the electron flux.

Gene expression and its regulation is described in a simplified fashion. We assume that the activity of the transcription factors ArcA and FNR depends on the metabolic signal quinone and oxygen, respectively. The change of oxidase activity and dehydrogenase activity depends linearly on the transcription factor activities.

In conclusion, the model combines physical constraints following from the balances of glucose, oxygen, biomass, intracellular electrons and the relevant enzymes with a model of the ETC regulatory circuits.

### Limitations of the model

The above mentioned simplifications limit the area of validity of the model. Firstly, the model is not intended for oxygen concentrations much higher than 100% aerobiosis because unmodelled mechanisms concerning oxygen stress might play a major role. Secondly, the reproduction of measurement data is best in the medium and upper micro-aerobic range. Whereas in the upper micro-aerobic range ubiquinone is the dominant quinone species, other quinone species (menaquinone and demethylmenaquinone) get increasingly important in the lower micro-aerobic range, [30]. Here, we compare the modelled quinone and quinol concentrations with measured ubiquinone and ubiquinol concentrations. Further, the different kinetics and energetics of the ETC enzymes in the lower micro-aerobic range is not reflected by the model. An extension of the model discriminating different quinone species, terminal oxidases and dehydrogenases will be presumably more accurate but will also require measurement data that discriminate the different terminal oxidases and dehydrogenases. Such data are currently not available. Despite those simplifications the model is able to reproduce and explain qualitative and quantitative differences between the experimental conditions, and it is sufficient for the further analysis.

### Differences between “ExpA” and “ExpB”

We used two different experimental conditions featuring two different wild-type strains of *E. coli*, Table 1. Both strains MC4100 and MG1655 are derivatives of K-12 with a functional electron transport chain and functional transcription factors FNR and ArcA. For MC4100, the functionality was shown, e.g. by [3]. The MG1655 strain used in our experiments originates from [46] and is able to express FNR, whereas other MG1655 strains were reported to have a large deletion around the *fnr* regulatory gene, [47]. The expression of ArcBA is supported by experimental data. One reason for differences between the two genotypes might be a reported genetic difference in the expression of *fnr*, [48]. In this publication the authors showed that some ETC genes have a changed expression because FNR transcript levels are reduced approximately fourfold in MC4100 (“ExpA”) compared to MG1655 (“ExpB”).

Measurement data for oxygen consumption, glucose consumption, fermentation product excretion and biomass concentration follow the same qualitative course for “ExpA” and “ExpB” (Fig. 4). The quantitative differences result from the different substrate concentrations in the inflow and the different dilution rate and are reproduced by the model. The electron pool e_2_H_2_ is fed by glucose uptake and drained by respiration, fermentation and growth, see Fig. 1. Since for a certain aerobiosis value the steady state substrate-biomass yield is fixed, the steady state glucose uptake is basically fixed by the growth rate (dilution rate) and the aerobiosis value. Under “ExpA” the respiration rate (Fig. 4C) is lower and the FNR activity (Fig. 6B) is higher than under “ExpB” because the higher glucose concentration in the inflow allows for a higher biomass concentration and thus less oxygen is available per cell.

Measurement data for ArcA activity and ubiquinone/ubiquinol concentration reveal a qualitative difference between “ExpA” and “ExpB”. The “ExpB” ArcA-P data show an almost monotonous decrease different to the one of “ExpA”, Fig. 5A. Secondly, the quinone redox state has different levels in the upper micro-aerobic range, see Fig. 5B. Finally, experimental condition “ExpA” leads to a constant total ubiquinone pool (reduced + oxidised) over the complete aerobiosis scale. In contrast, experimental condition “ExpB” is characterized by a strong increase of the total ubiquinone pool with the aerobiosis value (Fig. 5D). We expected further quantitative differences in the gene expression between “ExpA” and “ExpB” because different strains and dilution rates are used. Both differences may lead to alterations in gene regulation, especially in transcription factor activities.

### Model validation

The two different data sets “ExpA” and “ExpB” provide a mean for model validation. We divided the unknown parameters in two types: we assumed that most parameters (type 1), e.g. the ones describing reaction kinetics like glucose or oxygen uptake, are equal between “ExpA” and “ExpB”, see Table 3. Type 2 parameters related to TF-dependent gene expression and *de novo* synthesis of quinones are allowed to differ between “ExpA” and “ExpB”, see Table 4. We first identified the values of all unknown model parameters using “ExpA” data. Then we applied the identified values of type 1 parameters to describe “ExpB”, while parameters of type 2 were re-identified.

The quantities in Fig. 4 depend mainly on the parameters of type 1. The good description of the data from “ExpB” based on the model fitted to the data from “ExpA” justifies the assumption that parameters of type 1 have similar values between “ExpA” and “ExpB”. The quantities in Fig. 5 depend strongly on the *de novo* synthesis of quinones and gene regulatory parameters. The good reproduction of the data shows that the qualitative difference in the measurement data between “ExpA” and “ExpB”, for example transcription factor activities, can be explained by quantitative differences in parameters of type 2.

### Control of ArcBA by quinones

The activity of ArcA is controlled by the different quinones and quinols of the ETC. The relative importance of the different quinone species is subject to a current debate. Alvarez et al. [32] showed that ubiquinone deactivates ArcBA and menaquinol is necessary for reactivation of ArcBA. In contrast, the results of Bekker et al. [30] stressed the importance of ubiquinol for reactivation of ArcBA and menaquinone for inhibition of ArcBA upon a shift from anaerobic conditions to low-aerobiosis growth conditions. Further, Sharma et al. [31] showed that also oxidised demethylmenaquinone can inhibit ArcA activity.

Our mathematical model provides a possibility to analyse the contribution of quinone and quinol to the activity of ArcA. Assuming that the pair ubiquinone and ubiquinol is the dominant quinone species in the upper micro-aerobic region, the model can contribute to the discussion about the contribution of the different quinone species on the ArcA control. The model fitted by “ExpA” presents an intermediate result about the interplay of quinone and ArcA. In the simulation, the course of quinone inversely resembles the ArcA “zig-zag” course, see Fig. 5. The *total* concentration of quinone and quinol 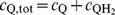 is virtually constant over aerobiosis. Therefore, this result cannot discriminate whether the quinone concentration alone *c*_Q_, the quinone redox state 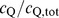 or some other combination of quinone and quinol concentration determines the observed ArcA activity. In contrast, the “ExpB” data set features a strong increase of the total concentration of quinone and quinol 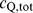, and we can discriminate between the hypothesis that the quinone redox state or the quinone concentration *c*_Q_ alone determines ArcA activity. A satisfactory simultaneous fit of the model to “ExpA” and “ExpB” is only possible with the assumption that oxidised quinone *c*_Q_ determines ArcA activity.

This result does not contradict the influence of ubiquinol reported by Bekker et al. [30] and Sharma et al. [31]. The model suggests that under the analysed experimental conditions the reactivation of ArcBA by quinol proceeds in saturation such that a variation of the concentrations of the reduced form has only a small effect on the reactivation rate of ArcBA. Then the activity of ArcBA results from the steady state between a deactivation rate that depends on the concentration of oxidised ubiquinone and an activation rate that is approximately constant. However a complete removal of reduced quinone species (for example by knock out mutations) will change the activation rate and thus the observed ArcBA activity. In conclusion, the model assumption that ArcA activity depends primarily on ubiquinone is valid for the *E. coli* wild-type in a steady state chemostat with varying oxygenation rates but not for experimental situations where the concentrations of ubiquinol is much lower.

The model does not consider the direct influence of other metabolites that are hypothesised signals for ArcA, as acetate, D-lactate, pyruvate or NADH, [28]. Model and measurement data consistently show that fermentation product excretion is higher under “ExpB” than under “ExpA”, because the metabolism is more reduced. However, the small difference as well as the courses of 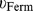 and ArcA activity suggest that fermentation products play a minor role in the modulation of ArcBA under the given experimental conditions. This is also consistent with Rodriguez et al. [49] who for the example of D-lactate state that it has a secondary level of control in comparison to quinones.

### Feed-forward motif in the regulation of the ETC

The model features two negative feedback loops: 1. Oxygen inhibits FNR and FNR inhibits oxygen uptake via the terminal oxidases. 2. Quinone inhibits ArcA and ArcA inhibits the quinone consumption via the dehydrogenases. These two regulatory feedback loops contribute to homeostasis of oxygen and quinone observed in the micro-aerobic range. The tight regulation is achieved by a sensitive dependence of the TF activities on their repressors. The values for the half-maximal activity constants 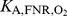 and 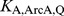 determine the magnitude of the concentration levels in the micro-aerobic range of oxygen and quinone, respectively. The two experimental conditions “ExpA” and “ExpB” differ in the available glucose concentration and thus reducing equivalents. Nevertheless, the regulatory effect of the sensitive negative feedback via ArcBA leads to a similar quinone concentration in the micro-aerobic range, (Fig. 5C).

Additionally to the inhibition of the oxidases, FNR also inhibits the expression of the dehydrogenases, [20]–[23]. Accordingly, an exclusive regulation of the dehydrogenases by ArcBA but not FNR cannot explain the “zig-zag” course of ArcA in “ExpA” (Fig. 5A): (i) the modelled mechanism between quinone concentration and ArcA activity is a bijective function, see equation (11b) as well as Fig. 3, hence both variables share the same monotony property (if quinone is monotonous, ArcA is monotonous and vice versa), (ii) an exclusive regulation by ArcBA on dehydrogenases (without FNR) would lead to a monotonous course of quinone over aerobiosis, and (iii) ArcA activity of “ExpA”, see Fig. 5A, is non-monotonous over aerobiosis. The measured and simulated “zig-zag” courses show a large drop in the lower micro-aerobic range, an increase of ArcA in the medium micro-aerobic range, a peak (lower than the maximal activity at anaerobiosis) and a drop around 100% aerobiosis. This observation conflicts with (i) and (ii). The characteristic ArcA activity was previously explained by the concerted influence of menaquinones and ubiquinones, [30]. Here, we show that this activity can also be explained by the influence of FNR on the expression of dehydrogenases. The parameter describing the strength of the feed-forward path (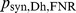) is consistently larger than the parameter describing the strength of the oxygen feedback path (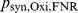), see Table 4. Following a change in oxygen concentration the dehydrogenase activity changes stronger than the oxidase activity. This leads to the seemingly paradoxical situation that additional oxygen input reduces the quinone pool in a steady state between 20% and 80% aerobiosis for “ExpA”, which is mirrored by an increase of ArcA activity. FNR feeds forward the information about oxygen availability to the ArcA regulatory loop.

**Figure 7:**
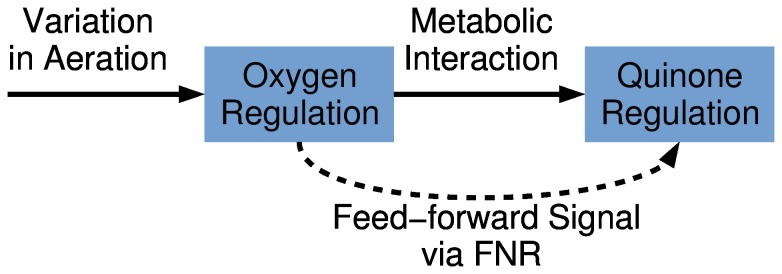
Signal flow upon a change of oxygen availability. The oxygen regulation system feeds forward the information about the oxygen availability via FNR to the quinone regulation system.

This design principle of a feed-forward loop, Fig. 7, is a common network motif in technical systems and in biological systems, e.g. transcription networks, [50]. By measuring a disturbance (here oxygen) and designing an appropriate feed-forward path (here via FNR) one can achieve a compensation or rejection of disturbance. The goal of a disturbance rejection is to increase the performance of the controlled system with respect to certain disturbances.

### Direction of electron flux

**Figure 8:**
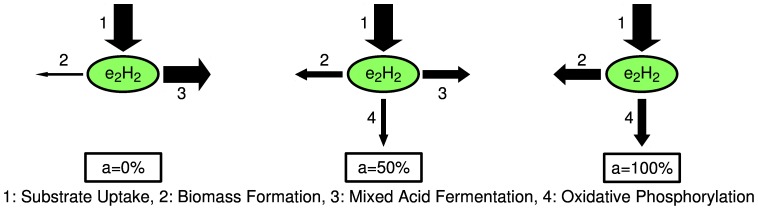
Electron distribution. The distribution of electrons (originating from substrate uptake) between biomass formation, mixed acid fermentation and oxidative phosphorylation changes for different aerobiosis *a*. The arrow sizes are proportional to simulated electron fluxes relative to the electron uptake flux.

The model elucidates the principles of how electrons are diverted under the studied experimental conditions. The only source of electrons is glucose uptake. Virtual all glucose provided to the reactor is consumed by the cells because glucose is the growth limiting substrate in the continuous culture and its residual concentration is marginal. Fig. 8 shows how the distribution of electrons between biomass formation, mixed acid fermentation and oxidative phosphorylation changes for different aerobiosis values. For increasing oxygen availability the electron distribution shifts from mixed acid fermentation to oxidative phosphorylation.

For aerobiosis values below 100%, the residual oxygen concentration is very low. This shows that the cell population virtually completely uses the provided oxygen. The rate of electrons required for growth is fixed because in a steady state chemostat the growth rate equals to the dilution rate. The biomass concentration increases approximately linear from its anaerobic value to its aerobic value, whereas the total oxygen consumption of the reactor is proportional to the aerobiosis value. This leads to the concave dependency of the specific oxygen uptake rate on the aerobiosis value (Fig. 4C). Fermentation product formation acts as a security valve to remove the excess of electrons from the metabolism. The specific fermentation rate has a convex dependency on the aerobiosis value (Fig. 4D) because the biomass-specific glucose uptake rate decreases and the respiration rate increases with growing aerobiosis value.

Around 100% aerobiosis multiple strong effects can be observed. The availability of the electron acceptor oxygen exceeds the availability of electrons from glucose uptake. In consequence, the oxygen concentration increases, Fig. 4A, while the excretion of fermentation products reaches zero, see Fig. 4D. Simultaneously, at 100% aerobiosis the quinone redox state drops, see Fig. 5B. This predicted drop was observed in measurement data presented by Bekker et al. [51]. The authors reported a quinone redox state value of 10% for comparable fully aerobic conditions in transition experiments. Neither acetate nor any other fermentation product is excreted in the fully aerobic state for the dilution rates discussed here.

However, for different experimental set-ups acetate “overflow metabolism” occurs even in the fully aerobic state, for example (i) for higher dilution rates (below the maximal growth rate) than used here [7], (ii) for certain *E. coli* mutants, e.g. Δ*sdhC* [8] or (iii) in batch experiments [8]. This overflow metabolism can occur, when all fluxes other than the formation of fermentation products have reached maximal capacity but substrate uptake still delivers electrons. In our simplified model, those limited fluxes would comprise the formation of biomass and the ETC (maximal activity despite available oxygen). In a more detailed perception, those fluxes must be further distinguished. For example, overflow metabolism might also occur by maximal citric acid cycle enzyme activities, because those reactions provide electrons for the ETC. However, our experimental conditions for this study were chosen such as to avoid aerobic overflow metabolism. For glucose-limited continuous cultures at dilution rates presented here, oxygen presents a limiting factor in the micro-aerobic range, whereas the lack of reducing equivalents becomes limiting at aerobic conditions.

### Potential applications

Previous investigations of micro-aerobic behaviour, e.g. [52], [53], were data-based while here, we present a mathematical model which not only provides a formalised description of the regulated ETC but also a new picture of underlying processes. Whereas Ederer et al. [36] focussed on a description of the metabolite concentrations and fluxes in the central metabolism, we highlight a simplified description of balancing electron donation and acceptance and the according regulation in *E. coli*.

An adaptation of the model to different conditions and strains is possible. For example, the adaptation to different electron acceptors and donors requires only marginal changes of the model. Also, the model can potentially be adapted to other organisms that share the FNR and ArcBA or homologous regulatory system (e.g. RegBA/PrrBA in *Rhodobacter* [54]). While maintaining the model structure, differences in growing conditions or strain must be accounted for by different parameter values of the respective pathways. Concurrently, experimental data from other strains and conditions can be used to validate our results.

Application areas where a thorough understanding of the regulation of the flow of redox equivalents is required include microbial production (e.g. hydrogen, glycerol or membranes with certain properties), bio-remediation (e.g. removal of heavy metals or waste water), food industry and medicine (e.g. host-pathogen-interaction or cancer therapy), see also for example [55]–[58]. Further, it is known that in aerobic large-scale reactors oxygen availability may be low in some regions due to bad mixing, [59]. The presented model can potentially provide insight into the re-organisation of metabolic fluxes in micro-aerobic regimes. For further modes of operation, data of transient experiments can be used to validate our results for the dynamic case.

## Conclusion

In summary, the *E. coli* oxygen response can be described by the proposed mathematical modelling approach. The general model behaviour is based on an electron balance. Electrons originate from an electron donor (glucose) and are either transferred to an electron acceptor (oxygen), excreted with fermentation products or incorporated into macromolecules. This model is coupled to a regulatory model of the electron transport chain, comprising the global transcriptional regulators ArcA and FNR. The mathematical model suggests that the regulatory structure of the ETC contains two feed-back loops assuring a homeostasis of oxygen via FNR and quinone via ArcA. A further central element of the regulation is a feed-forward motif for disturbance compensation of the influence of oxygen on the quinone-ArcA feed-back loop. For both presented experimental conditions, the mathematical model supports the hypothesis that for a wide range of aerobiosis values the ArcA activity depends mainly on the concentration of oxidised ubiquinone and that other known or suggested signals of ArcB, like menaquinol, are relevant only in the lower micro-aerobic range. Being integrated into a description of bacterial metabolism the model may provide the opportunity for a better control in a certain range of oxygenation, e.g. for optimising the production of organic compounds or for treatment of pathogens which prefer certain conditions.

## Materials and Methods

Experimental data result from glucose-limited continuous culture experiments at defined aerobiosis values thus exhibiting individual steady states. Measurements originate from two different experimental conditions and are available as Data S1. The differences between the two experimental conditions named “ExpA” and “ExpB” are listed in Table 1. Some data as well as their materials and methods were published previously, some data have been collected for this publication, see Table 2. For the former references and short summaries are given, while the latter are described in more detail. Further, a brief overview of modelling materials and methods is given.

### Bacterial strains and culture conditions

Chemostat experiments of different oxygen availability were carried out. The aerobiosis was defined from measurement of acetate formation rates following the aerobiosis scale definition of [4], see also [3], [5], [6]. The “ExpA” data originate from [3]–[6], [30]. The “ExpA” experiments were carried out with wild-type *E. coli* K-12 MC4100, at dilution rates of 0.15 h^−1^, applying glucose-limited medium (45 mM) with a temperature of 35°C and pH = 7. The strain used for “ExpB” was wild-type *E. coli* K-12 MG1655, [46]. For “ExpB”, chemostat experiments were carried out as described in [8], [44], [60] at dilution rates of 0.2 h^−1^, applying glucose-limited medium (20 mM) with a temperature of 37°C and pH = 6.9.

### Measurement of biomass, extracellular metabolite concentrations, quinones and ArcA phosphorylation status

Dry cell weight (DCW) of biomass was determined as described in [8]. A sample of culture broth was centrifuged, the pellet resuspendend in deionised water, again centrifuged and the DCW estimated from the dried pellet. Extracellular metabolite concentrations were determined as described in [44] and [8]. Oxygen uptake rate was determined by gas sensors. Extracellular metabolite concentrations of glucose and fermentation products were measured either by high-performance liquid chromatography (LKB) with a REZEX organic acid analysis column (Phenomenex), using an RI 1530 refractive index detector (Jasco) and AZUR chromatography software for data integration, as described in [44], or by enzyme kits and spectrophotometer, see [8]. Quinone extraction and analysis was performed as described in [60]. After a multi-step extraction procedure the quinone content was analysed by high-performance liquid chromatography and spectroscopy. ArcA phosphorylation status was determined as described in [60]. Phosphorylation was measured using Phos-tag-acrylamide gel electrophoresis and subsequent Western immunoblotting. Full activity was defined for 50% phosphorylation.

### Model Development and Simulation

The mathematical model was implemented, simulated and analysed within the MATLAB (The Mathworks, Natick, Massachusetts, United States) environment using the SBTOOLBOX2, [61]. The modelling work is discussed in the results and discussion sections in detail. The steady state chemostat measurement data are compared to quasi steady state results of a dynamic model excited by a slowly changing input of oxygen inflow 
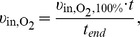
with 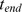 being an arbitrary large parameter. This definition is scaled by the oxygen inflow at 100% aerobiosis 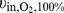, a parameter which can differ between experiments but which can be calculated from measurement data, see Text S2.

The free model parameters were identified using a particle swarm algorithm from SBTOOLBOX2, [62], minimizing the sum of squared errors between simulated and measured chemostat data.

## Supporting Information

Text S1 **Relation between electron pairs and fermentation products.**
(PDF)

Text S2 **Determination of parameter values using measurement data.**
(PDF)

Data S1 **Experimental data.**
(ZIP)
