# Supplementary material for: Basic Regulatory Principles of Escherichia coli's Electron Transport Chain for Varying Oxygen Conditions
Source: PLoS One. 2014 Sep 30;9(9):e107640. doi: 10.1371/journal.pone.0107640 (PMC4182436; doi:10.1371/journal.pone.0107640)
Supplement: Text S1 — Relation between electron pairs and fermentation products. (PDF) [file pone.0107640.s001.pdf]

Relation between electron pairs and fermentation products

The variable e_2H_2 describes a reducing or electron potential, i.e. its concentration represents available electron pairs, coupling glucose (electron donor) and electron acceptors, for example oxygen. This corresponds to an electron or redox balance, not a carbon balance.

The number of electron pairs available for respiration can be calculated by formulating the stoichiometric equation of total combustion. In total combustion all electron pairs of an electron donor are transferred to oxygen. An dioxygen molecule O_2 can accept 2 electron pairs. One molecule of glucose requires $6O_2$ for total combustion and thus carries 12 electron pairs:

$$C_6H_{12}O_6 + 6O_2 \rightarrow 6CO_2 + 6H_2O.$$

In a similar manner, also the electron pairs carried by fermentation products like acetate, ethanol, formate and lactate as well as succinate can be determined. This allows for calculating a total fermentation flux from measured fluxes of the respective individual species.

- Ethanol carries 6 electron pairs:

$$C_2H_5OH + 3O_2 \rightarrow 2CO_2 + 3H_2O.$$

- Acetate carries 4 electron pairs:

$$\begin{aligned} CH_3COOH + 2O_2 &\rightarrow 2CO_2 + 2H_2O, \\ C_2H_4O_2 + 2O_2 &\rightarrow 2CO_2 + 2H_2O. \end{aligned}$$

- Formate carries 1 electron pair:

$$\begin{aligned} CH_2O_2 + \frac{1}{2}O_2 &\rightarrow CO_2 + H_2O, \\ 2CH_2O_2 + O_2 &\rightarrow 2CO_2 + 2H_2O. \end{aligned}$$

- Lactate carries 6 electron pairs:

$$\begin{aligned} CH_3\text{-}CHOH\text{-}COOH + 3O_2 &\rightarrow 3CO_2 + 3H_2O, \\ C_3H_6O_3 + 3O_2 &\rightarrow 3CO_2 + 3H_2O. \end{aligned}$$

- Succinate carries 6 electron pairs:

$$C_4H_4O_4 + 3O_2 \rightarrow 4CO_2 + 2H_2O.$$

Aggregating these results, the total fermentation flux $v_{\text{Ferm,meas}}$ can be determined in dependence on the respective measured individual fluxes:

$$v_{\text{Ferm,meas}} = 6 \cdot v_{\text{Eth,meas}} + 4 \cdot v_{\text{Acet,meas}} + 1 \cdot v_{\text{Form,meas}} + 6 \cdot v_{\text{Lact,meas}} + 6 \cdot v_{\text{Succ,meas}}.$$

For “ExpA” the values for individual fluxes can be taken from [1]. At $a = (0, 23, 53, 100)\,\%$ the total fermentation flux can be calculated to $v_{\text{Ferm,meas}} = (64.2, 38.89, 14.54, 0.06)\,\text{mmol} \cdot \text{g}_{\text{DCW}}^{-1} \cdot \text{h}^{-1}$, see also respective Figure in main document.

References

1. Alexeeva S, de Kort B, Sawers G, Hellingwerf KJ, Teixeira de Mattos MJ (2000) Effects of limited aeration and of the ArcAB system on intermediary pyruvate catabolism in *Escherichia coli*. J Bacteriol 182: 4934–4940.
