# Supplementary material for: Basic Regulatory Principles of Escherichia coli's Electron Transport Chain for Varying Oxygen Conditions
Source: PLoS One. 2014 Sep 30;9(9):e107640. doi: 10.1371/journal.pone.0107640 (PMC4182436; doi:10.1371/journal.pone.0107640)
Supplement: Text S2 — Determination of parameter values using measurement data. (PDF) [file pone.0107640.s002.pdf]

Determination of parameter values using measurement data

- $Y_{0\%}$ and $Y_{100\%}$:
  The parameters $Y_{0\%}$ and $Y_{100\%}$, describing the glucose-biomass yield at 0 % and 100 % aerobiosis respectively, can be calculated from known glucose input and resulting steady state biomass for the anaerobic and aerobic case:

$$Y_{0\%} = \frac{D}{v_{\mathrm{Glc}}(0\%)} \approx \frac{c_{\mathrm{x}}(0\%)}{c_{\mathrm{in,Glc}}}$$
$$Y_{100\%} = \frac{D}{v_{\mathrm{Glc}}(100\%)} \approx \frac{c_{\mathrm{x}}(100\%)}{c_{\mathrm{in,Glc}}}$$

  These values equal to $Y_{0\%} = 0.02\,\mathrm{g_{DCW}} \cdot \mathrm{mmol}_{\mathrm{Glc}}^{-1}$ and $Y_{100\%} = 0.07\,\mathrm{g_{DCW}} \cdot \mathrm{mmol}_{\mathrm{Glc}}^{-1}$.

- X_{μ}:
  The parameter X_{μ} describing the proportion of electron pairs for forming biomass can be calculated from the state equation for $c_{\mathrm{e_2H_2}}$ using steady state measurement data and $v_{\mathrm{Oxi}} = v_{\mathrm{Dh}}$. Here we present the solution for the anaerobic and aerobic case only:
  anaerobic:

$$\begin{aligned} 0 &= 12 \cdot v_{\mathrm{Glc}}(0\%) - X_{\mu} \cdot \mu - v_{\mathrm{Ferm}}(0\%) \\ X_{\mu} &= \frac{12 \cdot v_{\mathrm{Glc}}(0\%) - v_{\mathrm{Ferm}}(0\%)}{D} \\ &= \frac{12}{Y_{0\%}} - \frac{v_{\mathrm{Ferm}}(0\%)}{D} \end{aligned}$$

  aerobic:

$$\begin{aligned} 0 &= 12 \cdot v_{\mathrm{Glc}}(100\%) - X_{\mu} \cdot \mu - v_{\mathrm{Oxi}}(100\%) \\ X_{\mu} &= \frac{12 \cdot v_{\mathrm{Glc}}(100\%) - v_{\mathrm{Oxi}}(100\%)}{D} \\ &= \frac{12}{Y_{100\%}} - \frac{v_{\mathrm{Oxi}}(100\%)}{D} \end{aligned}$$

  The calculated values were used for defining the lower and upper parameter bound for parameter identification. They result in $88.8\,\mathrm{mmol_{e_2H_2}} \cdot \mathrm{g_{DCW}^{-1}}$ and $112.8\,\mathrm{mmol_{e_2H_2}} \cdot \mathrm{g_{DCW}^{-1}}$, respectively.

- $v_{\mathrm{in,O_2,100\%}}$:
  The parameter $v_{\mathrm{in,O_2,100\%}}$ can be calculated using steady state data of biomass and oxygen uptake rate at 100 % aerobiosis:

$$v_{\mathrm{in,O_2,100\%}} = 0.5 \cdot v_{\mathrm{Oxi}}(100\%) \cdot c_{\mathrm{x}}(100\%)$$

  The value is different for the two experimental conditions resulting in values of $18.28\,\mathrm{mM_{O_2}} \cdot \mathrm{h}^{-1}$ for “ExpA” and $10.84\,\mathrm{mM_{O_2}} \cdot \mathrm{h}^{-1}$ for “ExpB”, respectively.

- p_{Glc}:
  This parameter can be calculated from the oxygen and glucose uptake. Indeed, in that way this parameter evaluated at 100 % aerobiosis ($\lambda_{\mathrm{Y}} = 1$) is related to $v_{\mathrm{in,O_2,100\%}}$:

$$\begin{aligned} p_{\mathrm{Glc}} &= \frac{v_{\mathrm{Oxi}}(100\%)}{v_{\mathrm{Glc}}(100\%)} = \frac{v_{\mathrm{Oxi}}(100\%) \cdot c_{\mathrm{x}}(100\%)}{c_{\mathrm{in,Glc}} \cdot D} \\ &= 2 \cdot \frac{v_{\mathrm{in,O_2,100\%}}}{c_{\mathrm{in,Glc}} \cdot D}, \end{aligned}$$

  resulting in a value of 5.42.
